# Keratin 17 modulates the immune topography of pancreatic cancer

**DOI:** 10.21203/rs.3.rs-3886691/v1

**Published:** 2024-02-20

**Authors:** Lyanne A. Delgado-Coka, Michael Horowitz, Mariana Torrente-Goncalves, Lucia Roa-Peña, Cindy V. Leiton, Mahmudul Hasan, Sruthi Babu, Danielle Fassler, Jaymie Oentoro, Ji-Dong Karen Bai, Emanuel F Petricoin, Lynn M. Matrisian, Edik Matthew Blais, Natalia Marchenko, Felicia D. Allard, Wei Jiang, Brent Larson, Andrew Hendifar, Chao Chen, Shahira Abousamra, Dimitris Samaras, Tahsin Kurc, Joel Saltz, Luisa F. Escobar-Hoyos, Kenneth Shroyer

**Affiliations:** Stony Brook University; Stony Brook University; Stony Brook University; Universidad Nacional de Colombia; Stony Brook University; Stony Brook University; Stony Brook University; Stony Brook University; Stony Brook University; Stony Brook University; Perthera Inc; Stony Brook University; Perthera Inc; Stony Brook University; University of Arkansas; Thomas Jefferson University; Cedars-Sinai: Cedars-Sinai Health System; Cedars-Sinai: Cedars-Sinai Health System; Stony Brook University; Stony Brook University; Stony Brook University; Stony Brook University; Stony Brook University; Yale University; Stony Brook University

**Keywords:** keratin 17, pancreatic ductal adenocarcinoma, cancer immunology, cancer biomarker, immune microenvironment, multiplexed immunohistochemistry, digital pathology

## Abstract

**Background:**

The immune microenvironment impacts tumor growth, invasion, metastasis, and patient survival and may provide opportunities for therapeutic intervention in pancreatic ductal adenocarcinoma (PDAC). Although never studied as a potential modulator of the immune response in most cancers, Keratin 17 (K17), a biomarker of the most aggressive (basal) molecular subtype of PDAC, is intimately involved in the histogenesis of the immune response in psoriasis, basal cell carcinoma, and cervical squamous cell carcinoma. Thus, we hypothesized that K17 expression could also impact the immune cell response in PDAC, and that uncovering this relationship could provide insight to guide the development of immunotherapeutic opportunities to extend patient survival.

**Methods:**

Multiplex immunohistochemistry (mIHC) and automated image analysis based on novel computational imaging technology were used to decipher the abundance and spatial distribution of T cells, macrophages, and tumor cells, relative to K17 expression in 235 PDACs.

**Results:**

K17 expression had profound effects on the exclusion of intratumoral CD8 + T cells and was also associated with decreased numbers of peritumoral CD8 + T cells, CD16 + macrophages, and CD163 + macrophages (p < 0.0001). The differences in the intratumor and peritumoral CD8 + T cell abundance were not impacted by neoadjuvant therapy, tumor stage, grade, lymph node status, histologic subtype, nor KRAS, p53, SMAD4, or CDKN2A mutations.

**Conclusions:**

Thus, K17 expression correlates with major differences in the immune microenvironment that are independent of any tested clinicopathologic or tumor intrinsic variables, suggesting that targeting K17-mediated immune effects on the immune system could restore the innate immunologic response to PDAC and might provide novel opportunities to restore immunotherapeutic approaches for this most deadly form of cancer.

## Introduction

Pancreatic ductal adenocarcinoma (PDAC) is one of the most lethal forms of cancer, not only because it is often not diagnosed until after it has reached advanced stage and is intrinsically resistant to Gemcitabine and 5-fluorouracil based chemotherapy, but because it generally does not respond to immune checkpoint inhibitors and is minimally impacted by intrinsic anti-tumor immune mechanisms (Muller et al., 2022). Although immune evasion is a key hallmark of malignancy, impacting cancer initiation and progression, knowledge of the mechanisms that shield PDAC from immune surveillance have not been fully explored. Therefore, elucidation of the interactions between PDAC and the immune response is critically needed to guide the development of more effective immunotherapeutic strategies.

Several studies have stratified PDAC patients into separate categories through transcriptomics, proteomic analysis, gene signatures or immunological status using bulk RNA-Seq, immunohistochemical, and single-cell RNA (scRNA) approaches (Y. Chen et al., 2022; de Santiago et al., 2019; J. Yang et al., 2022a). Although numerous transcriptomic and proteomic reports have shown that PDAC can be subdivided into major molecular subtypes that differ in response to chemotherapeutic agents and patient survival, little is known about how biologically distinct PDACs can differ in their immunogenic phenotypes, or the impact of the immune response on disease progression and survival. To the best of our knowledge, this is the first study that aims to consolidate the histological subtype stratification with the tumoral microenvironment status to better understand tumor aggression and rationalize more personalized therapeutic strategies. We and others have shown that keratin 17 (K17) drives chemoresistance and is a prognostic and predictive biomarker of the most aggressive (basal) molecular subtype of PDAC (Oblein et al., n.d.; C.-H. Pan, Chaika, et al., 2022; C.-H. Pan, Tseng, et al., 2022; Roa-Pena et al., 2019, 2021). K17 expression also impacts the immune response in several cancer types, including basal cell carcinoma, head and neck cancer (Depianto et al., 2010; Wang et al., 2022b, 2023), and cervical squamous cell carcinoma (Baraks et al., 2022). At a mechanistic level, K17 has also been reported to impact the pathogenesis of cervical squamous cell carcinoma, at least in part via immunomodulatory mechanisms (W. Wang et al., 2020b) and others have explored mechanisms through which K17 might regulate resistance to immunotherapy, through the regulation of Yap1 activation, mediating downstream immunosuppressive effects in head and neck cancer. The potential impact of K17 on the immune response to PDAC, however, has not been previously explored.

Thus, it is important to consolidate different stratification schemes into a novel classification of pancreatic cancer, based on robust and clinically deployable biomarkers to predict survival and to rationalize therapeutic strategies. Several studies have emphasized the importance of cancer cell clearing by intratumoral and peritumoral immune cells, with favorable prognosis related to the extent of intratumoral immune infiltration (Pyo et al., 2021). Since successful immunotherapy is dependent on the infiltration into the tumor of sufficient effector cells, including CD8 + T cells and tumor-associated macrophages, we aimed to characterize the PDAC immune microenvironment relative to K17 expression by focusing on peritumoral and intratumoral immune cells via a comprehensive, distance-based spatial analysis using brightfield multiplex immunohistochemistry (mIHC) of PDAC tissue sections. Overall, these lines of exploration may uncover how tumor cell-intrinsic immunomodulatory proteins, including K17, may shield PDAC from the development of effective immune responses and may highlight opportunities for further exploration to develop novel and more effective immunotherapeutic approaches for PDAC.

## Methods

### Patient demographics

Primary PDAC surgical resection specimens (n = 235) were provided as formalin-fixed paraffin-embedded (FFPE) surgical tissue blocks from the archival collections of the Department of Pathology at Stony Brook University Hospital (n = 54, 23%), Thomas Jefferson University (n = 67, 29%), Cedars Sinai Medical Center (n = 7, 3%) and a national biorepository, the Know Your Tumor program of the Pancreatic Cancer Action Network (PanCAN/Perthera) (n = 107, 45%).

Hematoxylin and eosin-stained sections from each specimen were reviewed to identify the single tissue block that contained the greatest total surface area of viable carcinoma. Exclusion criteria included cases where the total surface area of viable tumor was < 1 cm^2^. Additionally, tumors metastatic to the pancreas from other anatomic sites were also excluded. Survival and adjuvant therapy data was obtained from each respective institution’s registry. Case stratification was based on tumor stage, histologic subtype, and histologic grade. Tumor stage was assigned based on 8th edition American Joint Committee on Cancer (AJCC) criteria (Amin et al., 2017; Chun et al., 2018) and histopathologic grade was based on World Health Organization (WHO) criteria (Nagtegaal et al., 2020). Table 1 summarizes the demographic and clinicopathologic features of all cases.

### mIHC

Multiplexed immunohistochemistry (mIHC) was performed on a Discovery Ultra Auto Stainer (Roche/Ventana, Oro Valley, AZ), using VENTANA reagents according to the manufacturer’s instructions. Antibodies for CD4 (helper T cells), CD8 (effector T cells), CD16 (pan-macrophage), CD163 (M2 macrophage), pancytokeratin (panCK), and K17 were provided by Roche Diagnostics Corporation through a sponsored research agreement (RD005216). Multiple chromogens (Red: CD4+, Purple: CD8+, Yellow: CD16+, CD163-, Green: CD16+, CD163+, Teal: panCK+, and Brown: K17+) were deployed to enable multispectral imaging of diverse immune cell populations within the cancer microenvironment. Details of the mIHC protocol are outlined in **Supplementary Table 1**.

### Cell Detection and Classification

The ensemble of ColorAE and U-Net was developed previously^12^ for the detection and classification of cells in mIHC images. ColorAE is a deep autoencoder which segments stained objects based on color; U-Net is a convolutional neural network (CNN) trained to segment cells based on color, texture and shape. The two methods provide complementary information and are used together to predict K17 and cell types (Hasan et al., 2022). Each model is trained separately, and predictions from each model are combined in the inference phase to create multi-class masks. The multiplex segmentation ensemble is applied on patches of size 580 × 580 pixels extracted from whole slide images (WSIs) at 0.346 μm/pixel resolution. The patches are extracted from tumor bed regions that were manually annotated by pathologists. Multi-class masks are generated on patches using ColorAE and U-Net as described (Hasan et al., 2022).

### Dataset and Model Training

The training and validation datasets for the deep learning models were generated from 23 WSIs. Expert pathologists manually put labeled dots into the vicinity of each cell over 1000×1000-pixel tiles extracted from these 23 WSIs. To speed up the manual annotation process, we generated superpixel masks using simple linear iterative clustering (SLIC) with 6000 segments. SLIC groups pixels into superpixels based on their color and spatial proximity, using a k-means clustering approach.

### Model Validation and Experimental Setup

We carried out a quantitative evaluation of our detection and classification model as previously described (Hasan et al., 2022). In summary, we obtained the following F1 scores for our ensemble model – CD4 F1 = 0.92, CD8 F1 = 0.89, CD16 F1 = 0.77, CD163 F1 = 0.93, K17 + F1 = 0.98, K17- F1 = 0.99. An F1 score is a statistical measure used in classification tasks, combining precision and recall into a single metric by calculating their harmonic mean. We used a dropout rate of 0.3 in the U-Net and the following color concentration thresholds in the colorAE model: 0.7 for K17-positive, 0.1 for K17-negative, 0.1 for CD4, 0.1 for CD8, 0.1 for CD16, and 0.1 for CD163. We carried out computation using resources provided through the National Science Foundation digital cyberinfraestructure eXtreme Science and Engineering Discovery Environment (XSEDE) (Towns et al., 2014).

### Quantification of Tumor-Immune Cell Spatial Relationships

**Figure 1:**
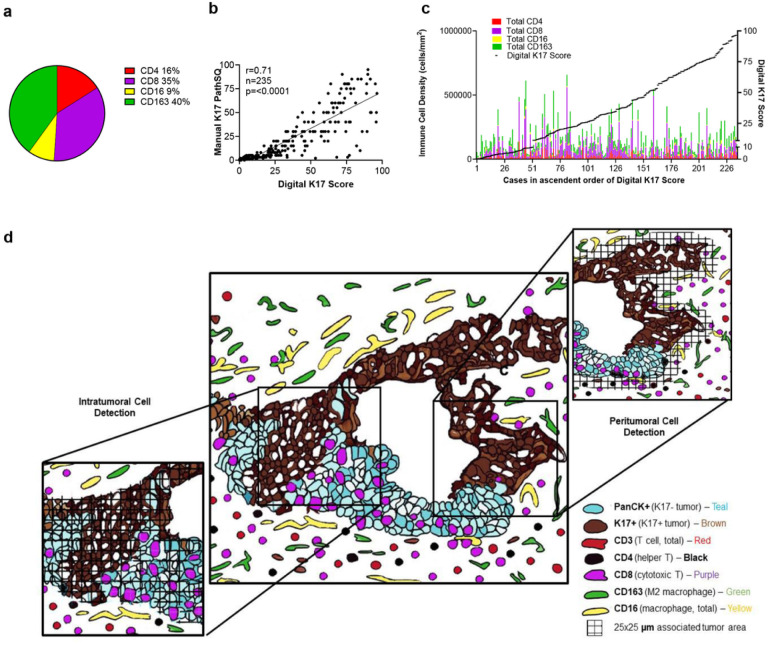
Analysis of Keratin 17 relative to the PDAC immune microenvironment. **a.** Overall fraction of immune cell types averaged across all cases (n=235). **b.** Spearman correlation between manual and digital K17 scoring across entire tumor sections. **c.** Overall immune cells stacked bar plot including CD4+ T cells, CD8+ T cells, CD16+ macrophage, and CD163+ macrophage density (cells/mm^2^). The right Y-axis depicts the overall K17 score within each tumor. **d.** Development of a digital scoring system focused on spatial relationships between peritumoral and intratumoral immune cells and K17. Intratumoral zones were defined as those that directly contacted a tumor cell while peritumoral zones included only immune cells within 25μm of the closest tumor cell boundary.

The tumor regions were partitioned into K17-positive and K17-negative zones, leveraging the masks generated with the ensemble model. Our goal was to compare immune cell density in regions close to K17-positive vs K17-negative tumor zones as well as intra-tumoral immune cell densities. First, we assessed the relative density of stromal immune cells in a range from 25 to 200um of the closest tumor border (defined as peritumoral immune cells) versus those that are in direct contact with K17-positive vs K17-negative tumor cells (defined as intratumoral immune cells). As the maximal differences in peritumoral immune cell counts relative to K17 status were seen at a stromal depth of 25μm the analsysi of all cases included in the study was done only at 25μm (**Supp**. Figure 1). In a conceptual sense, the approach we took was to associate each immune cell with K17-positive tumor cells when the closest tumor boundary to the cell was K17-positive and to associate immune cells with K17-negative tumor when the closest tumor boundary was K17-negative. The analysis described below formalizes this approach.

A distance transform mapped each pixel to the closest boundary of interest. We only considered stromal immune cells that are within 25μm of the closest tumor boundary; this region was computed using the distance transform (Fabbri et al., 2008; Strutz, 2021). We then partitioned this tumor-associated stromal region into K17-positive and K17-negative zones, leveraging the distance transform field of the stromal area. A stroma pixel was assigned to the K17-positive influence area when the closest tumor boundary was K17-positive, according to distance transform calculation; otherwise, the pixel was assigned to the K17-negative influence area. We devise a metric that we named the “Tumor/Stromal Zone Score”, denoted by $ZS_{M}^{ic}$, calculated by the following equation:

$$ZS_{M}^{ic}=\frac{\;{\text{CellCount}}_{M}^{i_{c}}}{\;\text{Tumor}/{\text{StromaZone}}_{M}}$$

In the equation,*i*_*c*_ represents immune cells (e.g., CD4, CD8, CD16 and CD163), and M represents the marker of tumori nest boundary (e.g., K17-positive boundary and K17-negative boundary). ${\text{CellCount}}_{M}^{ic}$ represents the number of immune cells of type *i*_*c*_ in either a K17 positive zone or in a K17 negative zone. The equation represents the approximate count of each immune cell (numerator) normalized by the total tumor-associated stromal zones (denominator). The estimation of immune cell count is achieved through a series of steps, commencing with the computation of total pixel numbers specific to distinct cellular subtypes. Following this, the pixel measurements were converted into square micron area units, subsequently undergoing normalization based on the average dimensions of immune cells. Notably, lymphocytes (CD4, CD8) average dimensions were approximated as circles with a diameter of around 8μm, while macrophages (CD16, CD163) average dimensions are approximated as circles with a larger diameter of 16μm. This normalization process culminated in the derivation of an estimated count of immune cells, designated as $\;{\text{CellCount}}_{M}^{ic}$ and represented in the Equation. In addition to calculating Tumor/Stroma Zone Score, we normalized Tumor/Stromal Zone Score for K17-negative $\left(ZS_{K17+}^{ic}\right)$ with respect to Tumor/Stromal Zone Score for K17-positive $\left(ZS_{K17+}^{ic}\right)$ for visualization and interpretation purposes, as depicted in multiple figures. Lastly, we performed proof of concept demonstrating that our observed pattern for Tumor/Stroma zone score for all the WSI is not random. We tested a statistical null hypothesis by randomly placing simulated immune cells in the tumor microenvironment and observed a statistically significant difference between the real and simulated scenarios, as previously reported (Hasan et al., 2022).

### Statistical analysis

Paired t tests were performed to define the difference between peritumoral and intratumoral immune cell counts in K17 positive and K17 negative regions of each case. Statistical significance was set at p-value ≤ 0.05, and analysis was done using SAS 9.4 (SAS Institute, Cary, NC, USA) and Graph Pad Prism 7 (Graph Pad Software, La Jolla, CA, USA). All p values were calculated using a two-sided test.

## Results

### Quantification of Tumor-Immune Cell Spatial Relationships Model

As the immune system is known to have a crucial role in cancer and play an essential role in eradicating tumor cells, the characterization of the immune component of the tumor microenvironment (TME) can provide valuable information regarding the ways in which the host immune response interacts with cancer cells (Karamitopoulou, 2019). We deployed mIHC and machine-learning tools to quantify T cells and macrophages in the tumor microenvironment relative to K17 expression by tumor cells across a broad range of clinically diverse PDAC cases (**Supp.** Figure 2.

### Overall Immune Cell Landscape in PDAC

The immune populations of 235 PDAC patients were processed by mIHC for a panel of myeloid and lymphoid cell markers encompassing CD8 + T cells, CD4 + T cells, CD16+/CD163- (M1) macrophages and CD16+/CD163+ (M2) tumor-promoting macrophages. Based on overall cell counts across all cases, 16% of immune cells were CD4 + T cells, 35% were CD8 + T cells, 40% were CD163 + macrophages, and 16% were CD16 + macrophages (respective mean counts 1.04 × 10^4^ /μm^2^, 3.00 × 10^4^ /μm^2^, 3.03 ×10^3^/μm^2^ and 2.44×10^4^/μm^2^) (Fig. 1a). To determine if the immune microenvironment was correlated with K17 status and to verify the accuracy of digital score, we confirmed that the K17 status based on a semi-quantitative manual scoring within a single representative histologic section from each case to K17 scoring based on image analysis of corresponding whole slide digital images (r = 0.71, p < 0.0001) (Fig. 1b). We then tested for correlations between the overall digital K17 score derived each tissue section to the immune cell counts for each case. Sorting patient’s immune densities in ascendant order of K17 expression revealed no obvious relationships at the macro level between K17 expression and any immune cell type (Fig. 1c).

Based on the premise that not only the relative abundance of T cells, but also the distribution and spatial relationship between T-cell subpopulations and cancer cells reflect biological interactions, we next set out to develop a model to score immune cells in the spatial context of direct interaction, reflected by immune cells that overlapped or directly contact tumor cells (intratumoral immune cells) versus those present within 25μm of the closest tumor cells (peritumoral immune cells), relative to the expression of K17 (Fig. 1d).

### K17 has profound effects on the PDAC immune microenvironment.

Analytic algorithms were developed to count intratumoral and peritumoral immune cells (respectively those that directly contact tumor cells versus stromal immune cells located within 25μm of the closest tumor cells, relative to K17 status). Immune cell counts were normalized relative to cell counts in K17-positive zones and results were ranked in order of increasing immune cell density ratios. In this analysis, immune cell ratios reflect differences in K17 negative versus K17-positive zones, rather than relative differences in overall immune cell counts across the entire tumor region.

**Figure 2:**
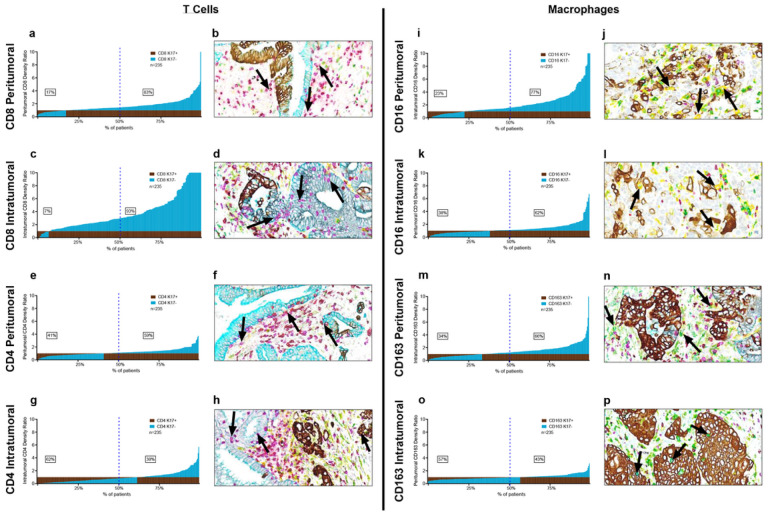
K17 impacts intratumoral and peritumoral T cells and macrophages. a-h. T cell counts in peritumoral and intratumoral K17-positive and K17-negative regions. **a.** Peritumoral CD8+ T cells. **c.** Intratumoral CD8+ T cells. **e.** Peritumoral CD4+ T cells. **g.** Intratumoral CD4+ T cells**. i-p.** Macrophage counts in peritumoral and intratumoral K17-negative regions relative to K17-positive regions. **i.** Peritumoral CD16+ macrophages; **k.** Intratumoral CD16+ macrophages; **m.** Peritumoral CD163+ macrophages; o. Intratumoral CD163+ macrophages. K17 expression profoundly excludes intratumor CD8+ T cells and to a lesser extent, peritumoral CD8+ T cells, CD16+ and CD163+ macrophages as noted by bargraph distribution (p<0.0001). Representative mIHC images for each panel highlight intratumor and peritumor **b,d.** CD8+ T cells (purple); **f, h.** CD4+ T cells (red); **j, l.** CD16+ macrophages (yellow) and; **n, p.** CD163+ macrophages (green) relative to K17-positive tumor cells (brown) and K17-negative tumor cells (teal). Note that immune cell ratios are normalized to counts in K17-positive zones and relative height of the bars reflects the magnitude of differences between ratios in K17-negative versus K17-positive zones, not relative differences in overall immune cell counts.

Cytotoxic T cells target tumor cells that expose tumor-specific antigens in various malignancies, including pancreatic ductal adenocarcinoma(Carstens et al., 2017; Masugi et al., 2010; Raghavan et al., 2021) and higher CD8 + T-cell density in tumor is generally associated with prolonged pancreatic cancer survival (Z. gang Chen et al., 2021; Li et al., 2022; Tsujikawa et al., 2017; B. Yang et al., 2021). Conversely, K17 has been associated with immune cell response in psoriasis as well as in basal cell skin cancer and in cervical carcinoma and is a negative prognostic biomarker in PDAC, suggesting that K17 might have some role in CD8 + T cell exclusion (Xiao et al., 2020; Zhou et al., 2022). Thus, to test for relationships between K17 expression the tumor inflammatory microenvironment, we analyzed intratumoral and peritumoral CD8 + T cells, CD4 + T cells, CD16+/CD163- tumor-targeting (M1) macrophages and CD16+/CD163 + tumor promoting (M2) immune cells ratios across all cases. CD8 + peritumoral T cells were more numerous in K17-negative areas than in K17 + areas *p* < 0.0001) in 83% of PDACs (Fig. 2a). Even more profoundly, intratumoral CD8 + T cell ratios were greater in K17-negative regions than in K17-positive regions in 93% of PDACs (*p* = < 0.0001) (Fig. 2c). Although the magnitude of the correlation with K17 was much less than seen for CD8 + T cells, peritumoral CD4 + T ratios were also greater in K17 negative areas for 59% of cases (Fig. 2e) but were increased in K17 + intratumoral areas in 62% of cases (Fig. 2g).

To uncover any relationships between K17 expression and macrophage distribution, we then analyzed the immune cell density of CD16 + macrophages and CD163 + macrophages across all cases. CD16 + cells were more abundant in K17 negative versus K17 positive peritumoral areas in 77% of cases (p < 0.0001) (Fig. 2i). Intratumoral CD16 + macrophages were more numerous in K17-negative tumor zones compared to the K17-negative regions in 62% of cases (p < 0.0001) (Fig. 2k). In peritumoral zones, CD163 + macrophages were more abundant in K17-negative zones in 66% of cases (p < 0.0001) (Fig. 2m). Conversely, intratumoral CD163 + macrophages were more numerous in K17-positive zones in 57% of cases (*p* = < 0.0001) (Fig. 2o). The relationships between CD16 + and CD163 + macrophages and K17 expression were independent of other clinicopathologic features, including tumor grade, pathological stage, treatment history, histologic variant, and mutational status (data not shown

**Figure 3:**
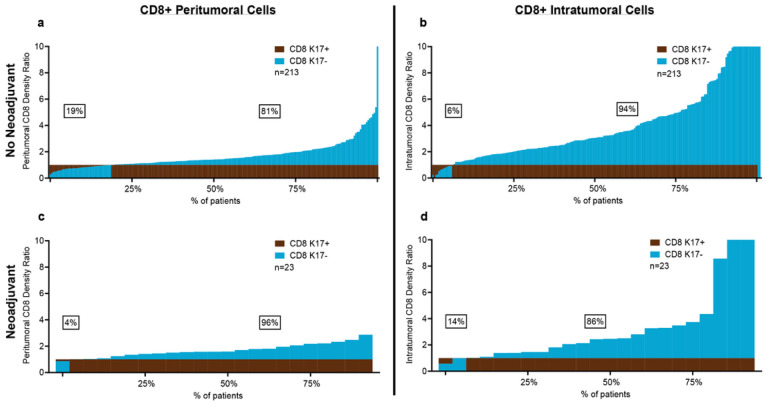
The impact of K17 on CD8+ T Cells is independent of neoadjuvant therapy. **a-b.** Peritumoral and intratumoral CD8+ T cell density ratios in cases that did not receive neoadjuvant treatment and, **c-d.**Cases treated with neoadjuvant treatment.

To explore changes in tumor-infiltrating immune cells in PDACs after neoadjuvant immunotherapy we separate our cohort into two categories, including patients that received gemcitabine-based or 5-FU based neoadjuvant treatment (n = 23, 10%) versus those that did not receive any neoadjuvant treatment before surgery (n = 212, 90%). CD8 + T cell ratios were consistently greater in K17-negative peritumoral and intratumor zones, for both no-neoadjuvant and neoadjuvant treatment groups (Fig. 3a–d). These results suggest that neoadjuvant therapy has minimal impact on CD8 + T cell ratios in K17-negative versus K17-positive tumor zones.

**Figure 4:**
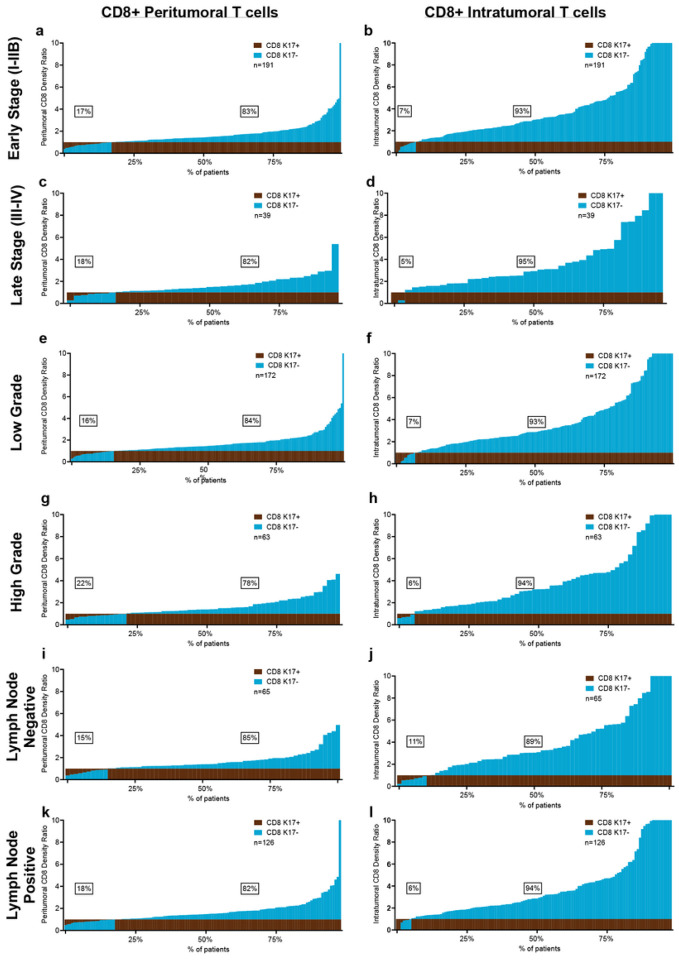
The impact of K17 on CD8+ T cells is independent of PDAC stage, grade, and lymph node status. Immune cell ratios in peritumoral and intratumoral K17-negative regions relative to K17-positive regions, ordered based on the density of immune cells in K17-positive zones. The inverse correlation between K17 expression and CD8+ peritumor and intratumoral T cells is independent of **a-d.** Stage, **e-h.** Tumor grade, and **i-l.** Lymph node status.

We next tested for relationships between tumor stage, grade and lymph node status and found that the inverse correlations between K17 + expression and CD8 + T cells are independent of each of these tumor-specific clinicopathologic variables (Fig. 4). Furthermore, CD8 + cell counts relative to K17 status were independent of tumor histologic subtype, including conventional, foamy cell, and large duct PDAC variants (**Supp.** Figure 3).

**Figure 5:**
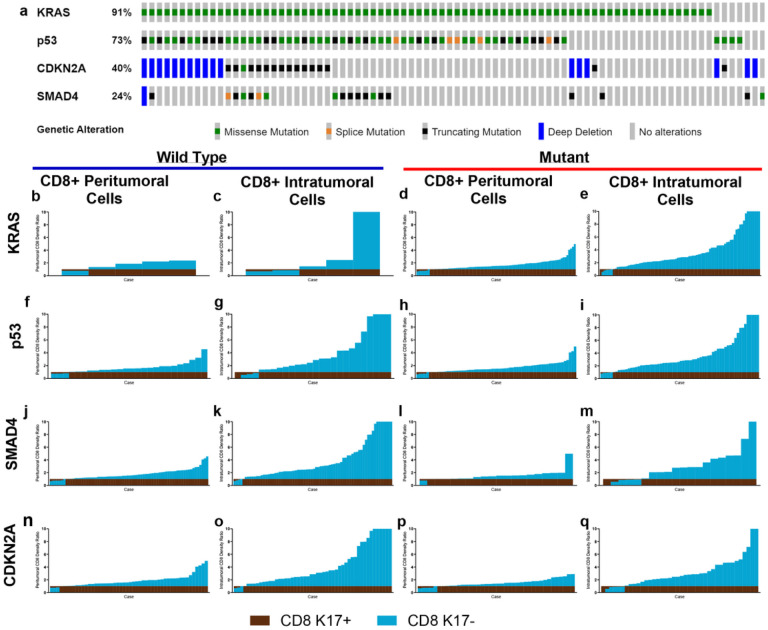
CD8+ T cells are increased in K17-negative regions, regardless of mutation status. Immune cell ratios in peritumoral and intratumoral K17-negative regions relative to K17-positive regions and mutational status of KRAS, p53, SMAD4, and CDKN2A. **a.** OncoPrint (Cerami et al., 2012; de Bruijn et al., 2023; Gao et al., 2013) depicting the most frequently mutated genes in the KYT cohort. **b-q.** Wild type versus mutant KRAS, p53, SMAD4, and CDKN2A.

Several studies have reported that TP53 missense mutations lead to reduce the infiltration of cytotoxic CD8 + T cells and approximately 70% of all PDACs harbor TP53 gene mutations (Maddalena et al., 2021; McCubrey et al., 2022; M. Pan et al., 2023). Furthermore, wild-type (WT) and mutant variants of p53 can modulate the antigen presentation machinery and can influence cytokine and chemokine secretion from the cancer cells, thereby impacting the immune TME (Maddalena et al., 2021). We set out to elucidate the impact of the 4 most common mutations on the immune TME of PDAC based on the analysis PDACs from the KYT cohort that had undergone comprehensive genomic sequencing through the Precision Promise program of the Pancreatic Cancer Action Network (Pishvaian et al., 2018, 2020) (Fig. 5a). We divided our samples based on their genomic status into WT or Mutant for each gene and we found that regardless of the mutational landscape, the impact of K17 CD8 + T cell rations within the immune microenvironment was unchanged (Fig. 5b–q).

Thus, K17 expression correlates with major differences in the immune microenvironment, most notably through profound exclusion of CD8 + T cells that is independent of clinicopathologic features or tumor intrinsic variables, treatment history, tumor grade, pathological stage, lymph node status, histologic variant, and tumor mutational status.

## DISCUSSION

Although K17 expression impacts gene expression, cell proliferation, and numerous other hallmarks of cancer, the impact of K17 on the immune response to PDAC has not previously been explored. In this study, we found that tumor cell expression of K17 expression impacts the PDAC microenvironment by shielding tumor cells from CD8 + T cells responses, while recruiting tumor promoting CD163+ (M2) macrophages, indicating that K17 impacts the immune response as a fundamental hallmark of aggression in PDAC. This work also provides a platform for image analysis of multiplexed immunohistochemical protocols that can efficiently analyze the immune composition of the cancer microenvironment.

PDAC is generally regarded as a “cold tumor” with a low T cell infiltration and low tumor mutation burden (TMB) with few neoantigens (Ullman et al., 2022). This makes the successful application of immunotherapy a very challenging task. High levels of T cell infiltration, however, correlate with improved outcome in PDAC (Goulart et al., 2021; Kiryu et al., 2021), including CD8 + T Cells (Orhan et al., 2020). Interestingly, the proximity of CD8 + T cells to tumor cells in the PDAC TME correlates to longer patient survival (Carstens et al., 2017). Consistent with our previous works which showed that K17 expression in PDAC is associated with shorter survival (Roa-Pena et al., 2021a; Roa-Peña et al., 2021b), our current findings also support the hypothesis that K17 blocks immune cell infiltration, with the most profound impact being on CD8 + T cells.

A multiparameter analysis of the immune landscape in PDAC revealed heterogeneous expression of immune checkpoint receptors in individual patients’ T cells and increased markers of CD8 + T cell dysfunction in the disease stage (Steele et al., 2020). *In vivo* studies have also shown that blockade of IL-1β increased the numbers of tumor-infiltrating lymphocytes and CD8 + T-cell responses. Furthermore, Wang et al. 2022 studied the role of K17 in cancer metastasis using an immunocompetent mice model and their results suggest that K17 confers resistance to immunotherapy. One mechanism through which K17 downregulates T cell infiltration could be through the suppression of CXCL9 production in macrophages through tumor cell-macrophage interactions. Other *in vivo* studies, also suggest that K17 expression suppressed T cell infiltration and enhanced neutrophil infiltration in in the tumor microenvironment of cervical cancers (Wang et al., 2023).

The delicate balance between the populations of CD4 + and CD8 + subsets determines whether the TME is anti- or pro-tumorigenic (Saka et al., 2020). Notably, regulating the differentiation of naïve CD4 + T cells into Th1, Th2, Th17, Th9, Th22, and Tregs is essential for eliminating immunosuppressive restrictions from the tumor environment and boosting effector T-cell activity (Huber et al., 2020; Karamitopoulou, 2019; Murakami et al., 2019). It is possible that the disruption of the correct ratio of these cell populations causes immune evasion in cancer and even the failure of several immune cell targeted therapies. We hypothesize that most CD4 T cells associated with K17-positive tumor areas are Tregs and that K17 contributes to PDAC growth by suppressing T cell infiltration. Although the mIHC panel described in this paper was not designed to identify CD4 T cells subsets, further studies to identify CD4 T cell subsets and their association with K17 expression in PDACs are ongoing in our lab.

K17 has a wide range of effects on the immune response in different tissues. For example, increased K17 expression upregulates the expression of multiple proinflammatory cytokines and chemokines, including IFN-γ, IL-22, and CXCL1, and plays an important role in the development of psoriasis. Whereas in models of head and neck cancer, the knockout of K17 gene expression slowed tumor growth and increased CD8 + T cell infiltrate in immunocompetent syngeneic C57/BL6 mice compared to parental MOC2 tumors(Rickman et al., 2008; Wang et al., 2022a). Here, we observed an inverse correlation between K17 and CD8 + T cells, as reported previously in other skin and allergic disease processes. Insight into the mechanism that underlie these effects may be inferred from previous studies that have linked K17 and CD8 + T cells in psoriasis and allergic contact dermatitis (ACD) (Luo et al., 2022; Xiao et al., 2020). Providing further insight into the mechanisms through which K17 acts in ACD, it was found that K17 translocates into the nucleus of activated keratinocytes, facilitating activation of STAT3 and downstream CCL20 production as well as T cell trafficking. Our lab previously reported that the soluble form of K17 undergoes nuclear translocation and serves as a nuclear shuttle of p27 (Escobar-Hoyos et al., 2015). Thus, it is possible that similar mechanisms may have a role in the immune response to PDAC. M2 macrophages contribute to chronic inflammation, cancer cell stemness, desmoplasia, immune suppression, and metastasis in PDAC, highlighting their importance in pancreatic cancer (Poh, 2021). Our observations that CD163+ (M2) macrophages are more numerous in K17-positive intratumoral areas are consistent with previous studies in colorectal cancer(Xue et al., 2021) and align with work depicting CD163 CD + T cells as promoter of biologic aggression in pancreatic cancer (J. Yang et al., 2022b).

In conclusion, our data support the hypotheses that K17 shields tumor cells from CD8 + T cells and recruits tumor promoting CD163 + M2 macrophages, indicating that K17 fundamentally impacts the immune response to PDAC. These effects are independent of neoadjuvant treatment, clinical pathologic features, or PDAC mutational status, suggesting that the interactions between K17 and immune cell responses in cancer are robust and could be important in both early stage and advanced stage disease. Beyond our exploration of tumor and immune cell interactions that are impacted by K17, the development of a platform for image analysis of multiplexed immunohistochemical protocols may also be applicable for the analysis of immune composition for solid tumors of other anatomic sites. Further studies are still needed to uncover how K17 expression facilitates evasion from immune surveillance, and to identify new druggable targets, relative to K17 status, that could enhance the efficacy of immunotherapy for PDAC. Whether K17 could also be used as a biomarker to identify subgroups of PDAC patients who may benefit from immunotherapy or could be therapeutically targeted to restore the efficacy of the innate immune response against PDAC should also be subjects of future research.

### Conclusions

K17 expression shields tumor cells from CD8 + T cells and recruits tumor promoting CD163 + M2 macrophages, indicating that K17 fundamentally impacts the immune response to PDAC. These effects are independent of neoadjuvant treatment, clinical pathologic features, or PDAC mutational status, suggesting that the interactions between K17 and immune cell responses in cancer are robust and could be important in both early stage and advanced stage disease. Therefore, targeting K17-mediated immune effects on the immune system could potentially restore the innate immunologic response to PDAC and might provide novel immunotherapeutic approaches for this devastating disease.

## Figures and Tables

**Table 1:** Patient cohort demographics

	Cohort
**Total cases included**	n = 235
**Overall survival, mean ± SD**	20.7 ± 17.1
**Age at diagnosis, mean ± SD**	62.9 ±14.3
**Gender, number (%)**		
Female	109 (46%)
Male	123 (52%)
Unknown	3 (2%)
**Histologic Grade (G), number (%)**		
G1 +G2, Well and Moderately Differentiated	172 (73%)
G3, Poorly Differentiated	63 (27%)
**AJCC 8th Edition Pathological Stage, number (%)**		
I-IIB (Early)	65 (27%)
III-IV (Advanced)	165 (70%)
Unknown	5 (3%)
**Chemotherapy**		
Neoadjuvant	41 (17%)
No Neoadjuvant	194 (83%)
**Histologic Subtypes**		
Conventional	180 (77%)
Foamy Gland (Adsay et al., 2000)	20 (9%)
Large Duct (Sato et al., 2021)	18 (8%)
Other	17 (6%)
**Genetic Mutation Status**		
KRAS, p53, SMAD4, CDKN2A	90 (38%)

## Data Availability

The datasets generated and/or analyzed during the current study are not publicly available due to IRB regulations but are available from the corresponding author on reasonable request. All required pipelines for digital image processing and related computational manuals are available at https://github.com/SBU-BMI/shroyer_lab_workflow to expand biomarker-based discovery and deployment in oncoimmunology research and improved ability to stratify and monitor patients receiving diverse immune based therapeutics.

## Abbreviations

ACD: Allergic contact dermatitis
AJCC: American Joint Committee on Cancer
CITI: Collaborative Institutional Training Initiative
CNN: Convolutional neural network
FFPE: Formalin-fixed paraffin-embedded
IRB: Institutional Review Board
K17: Keratin 17
mIHC: Multiplexed immunohistochemistry
PanCAN: Pancreatic Cancer Action Network
panCK: Pancytokeratin
PDAC: Pancreatic ductal adenocarcinoma
ROI: Region of interest
scRNA: Single-cell RNA
SLIC: Simple linear iterative clustering
TMB: Tumor mutation burden
TME: Tumor microenvironment
WHO: World Health Organization
WSI: Whole slide image
WT: Wild type
XSEDE: eXtreme Science and Engineering Discovery Environment
